# Exercise Stress Test–Induced Atrioventricular Dissociation With Syncope

**DOI:** 10.31486/toj.20.0134

**Published:** 2021

**Authors:** Zin Thawdar Oo, Dishang Bhavsar, Thin Phyu Phyu Aung, Cesar E. Ayala-Rodriguez, Htoo Kyaw

**Affiliations:** ^1^ University of Medicine 1, Yangon, Myanmar; ^2^ Department of Internal Medicine, Jacobi Medical Center/North Central Bronx Hospital, Bronx, NY; ^3^ Department of Internal Medicine, The Brooklyn Hospital Center, Brooklyn, NY; ^4^ Academic Affiliate of The Icahn School of Medicine at Mount Sinai, New York, NY; ^5^ Clinical Affiliate of The Mount Sinai Hospital, New York, NY; ^6^ Department of Internal Medicine, University of Mandalay, Mandalay, Myanmar; ^7^ Division of Cardiology, The Brooklyn Hospital Center, Brooklyn, NY; ^8^ The Zena and Michael A. Wiener Cardiovascular Institute, Icahn School of Medicine at Mount Sinai, New York, NY

**Keywords:** *Atrioventricular dissociation*, *chest pain*, *heart block*

## Abstract

**Background:** The exercise stress test is widely used as a diagnostic test for evaluating coronary artery disease in symptomatic patients or those with underlying cardiovascular disorders. Although exercise stress test risk is minimal with a <1% chance of causing heart block, physician awareness of potential complications is paramount for prompt recognition and treatment.

**Case Report:** A 65-year-old-female with angina-like chest pain underwent an exercise stress test for ischemic heart disease evaluation. She performed the exercise stress test up to stage 2 (exercise Bruce protocol) with an exercise duration of 5 minutes and maximum metabolic equivalents of 7. During her recovery phase, the patient developed atrioventricular dissociation with junctional rhythm followed by syncope. Immediate treatment was administered, including intravenous normal saline, and she recovered without any complications.

**Conclusion:** This case reminds clinicians to be aware of the unpredictable effects of the exercise stress test even though atrioventricular dissociation after an exercise stress test is rare. Providing immediate treatment to prevent any untoward effects is essential.

## INTRODUCTION

Many physiologic changes are associated with exercise, such as heightened sympathetic tone, increased heart rate (HR), and myocardial oxygen consumption.^1^ American cardiologist Robert A. Bruce described the use of the Bruce protocol treadmill stress test in 1963 to assess cardiovascular function.^2^ Bruce's first reports on the treadmill exercise test in 1949 evaluated intrinsic physiologic changes in the respiratory and circulatory function of normal adults, as well as in patients with heart and lung diseases.^3,4^ Although the exercise stress test is generally safe and is principally used as a diagnostic test for evaluating coronary artery disease (CAD) in symptomatic patients, the test is associated with some rare adverse effects.

Some people may experience shortness of breath, chest pain, dizziness, and collapse, especially if they have underlying comorbidities such as asthma, chronic obstructive pulmonary disease, conduction abnormalities, and heart failure.^5^ However, electrocardiogram (ECG) treadmill testing is recommended as the first choice for patients with a medium or high risk of CAD because few resources are required, and the test is low cost with no radiation exposure. The risk of CAD can be estimated based on the presence of risk factors such as smoking, hypertension, diabetes mellitus, hyperlipidemia, and family history of CAD.^6^

We present the case of a patient with angina-like chest pain who performed an exercise stress test and experienced syncope secondary to exercise-induced heart block.

## CASE REPORT

**Figure 1.**
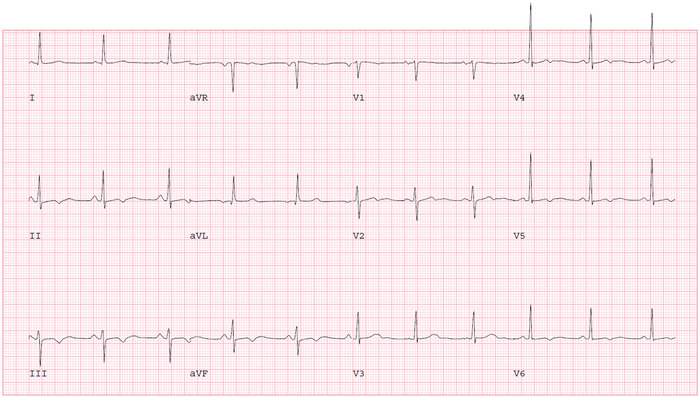
Resting electrocardiogram shows sinus bradycardia with nonspecific T wave changes.

A 65-year-old-female with a medical history of hypertension and diabetes mellitus presented to the emergency department with left-sided chest pain for 3 days. The pain was located in the midline and characterized as pressure-like, nonradiating, sometimes worse with ambulation, and occasionally relieved by rest. During the first encounter, the patient was afebrile and had blood pressure (BP) of 172/92 mmHg with an HR of 49/min, respiratory rate (RR) of 16/min, and oxygen saturation of 98% on room air. ECG revealed sinus bradycardia with HR of 54/min and nonspecific T wave changes in the inferior leads (Figure 1). Blood tests revealed brain natriuretic peptide of 85 pg/mL (reference, <100 pg/mL) and thyroid-stimulating hormone of 0.69 mU/L (reference range, 0.5-5.0 mU/L), excluding heart failure and hypothyroid induced bradycardia. Myocardial infarction was ruled out by serial negative troponin tests. Because the patient had uncontrolled hypertension upon admission, medical optimization with losartan 50 mg daily and nifedipine ER 30 mg daily for better BP control was performed. Transthoracic echocardiogram showed left ventricular ejection fraction of 60%-65% with no significant valvular heart disease.

**Figure 2.**
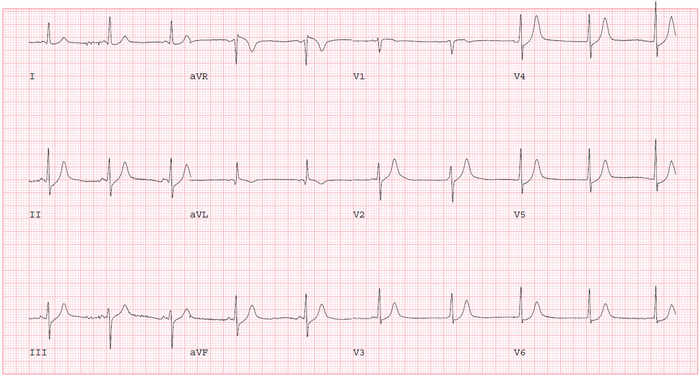
During the recovery phase at 1 minute 19 seconds, junctional rhythm replaced sinus rhythm.

**Figure 3.**
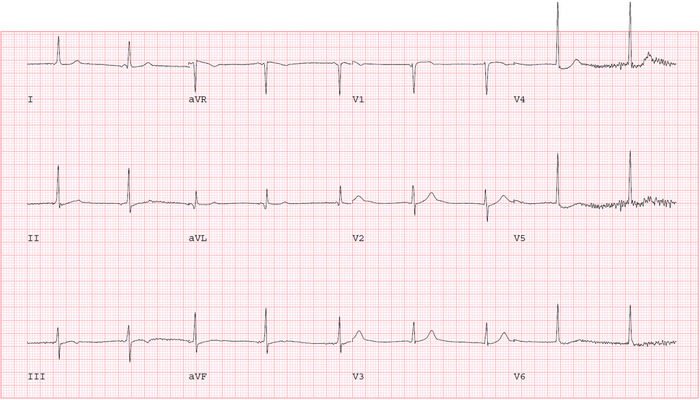
Junctional and sinus rhythm can be seen competing with each other during recovery stage three, 2 minutes 50 seconds after exercise.

**Figure 4.**
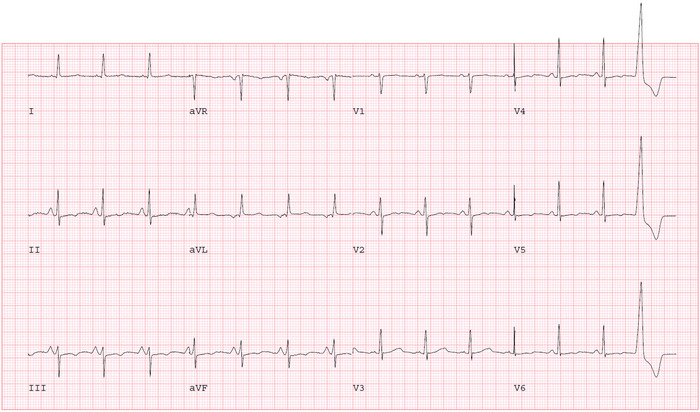
Sinus rhythm was reestablished with premature ventricular contraction at the end of recovery stage six, 5 minutes 50 seconds after exercise.

The following day, the patient reported some improvement in her chest pain. Given the presence of CAD risk factors with angina-like chest pain, further testing for ischemic heart disease evaluation was pursued. She performed an exercise Bruce protocol up to stage 2 with an exercise duration of 5 minutes, maximum metabolic equivalents of 7, and a maximum HR of 141/min (90% of maximal, age-predicted HR). Prior to starting the exercise stress test, her vital signs were BP of 145/75 mmHg and HR of 65/min. During the recovery phase, the patient developed an atrioventricular (AV) dissociation episode with a junctional rhythm at 1 minute and 19 seconds (Figure 2). Her HR was 60/min, BP was 90/60 mmHg, and RR was 18/min with oxygen saturation of 98% on room air. The patient reported dizziness and sweating. We immediately laid her down on the table and started intravenous (IV) fluid with normal saline while closely monitoring her HR. Telemetry monitoring showed the lowest HR was 54/min with competing junctional rhythm at recovery stage three, 2 minutes 50 seconds after exercise (Figure 3). The patient then experienced a transient syncopal episode for a few seconds. She began feeling better with the IV saline infusion, and ECG revealed that sinus rhythm with occasional premature ventricular contraction took over the junctional rhythm at recovery stage six, 5 minutes 50 seconds (Figure 4).

**Figure 5.**
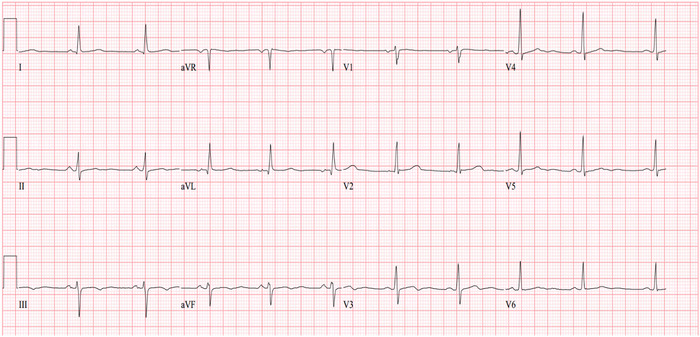
The day after the stress test, electrocardiogram showed normal sinus rhythm without conduction abnormality.

She was feeling better on the following day without any focal weakness but still experienced intermittent chest pain. Given the presence of residual chest pain with transient syncope, she underwent coronary angiography that showed nonobstructive CAD with a 30% ostial lesion in the left anterior descending second diagonal artery. The next day, ECG and continuous telemonitoring showed no heart block, pauses, or junctional rhythm (Figure 5). Because of the isolated bradycardic event after exercise, we decided to perform additional continuous cardiac monitoring with a loop recorder rather than placing a permanent pacemaker. The patient was discharged home without any further events on day 4 of hospitalization, along with guideline-directed medical therapy of losartan 50 mg daily, nifedipine ER 30 mg daily, atorvastatin 40 mg daily, and metformin 500 mg twice a day. She was seen in the outpatient cardiology clinic without any symptoms 1 week later and 2 to 3 months thereafter.

## DISCUSSION

Exercise-related conduction abnormalities are not uncommon in the literature, as different types of heart blocks, including right bundle branch block (RBBB), left bundle branch block (LBBB), and hemiblock, can develop during the exercise stress test.^7^ The development of rate-dependent LBBB during an exercise stress test is one of the well-known and studied phenomena.^8^ First-degree AV block or the progression of preexisting AV block to advanced AV block can sometimes be found in association with exercise, although syncope is a rare event during exercise stress test.^1^ However, in a study by Cerqueira et al, 7.6% of patients undergoing a nuclear stress test with adenosine developed an AV block of any type; 0.78% developed third-degree AV block.^9^

AV nodal heart block can be divided into 2 categories based on the location: AV nodal and infranodal block. AV nodal conduction, regulated by the autonomic nervous system, is modulated by changes in sympathetic activities during exercise.^10,11^ If the heart block improves with exercise, the pathology is likely at the AV node level, whereas the disease can be at the infranodal area level if it worsens.^12^ The mechanism for exercise-induced AV dissociation is not clearly understood. One hypothesis is that the AV node refractory period decreases during exercise while the His-Purkinje system remains unaffected. In a normal conduction system, this difference may not affect the accelerated conduction during exercise but may appear as AV block or nonconducted P waves with exertion in a diseased conduction system.^13,14^ Another proposed mechanism is that the occurrence of AV nodal ischemia during exercise could manifest as an AV block.^13^ Sumiyoshi et al studied the clinical and electrophysiologic phenomena of exercise-induced AV block in 14 patients and noticed that AV block can occur at any level of the AV conduction system; however, they found no clear etiology of AV exercise-induced AV block.^15^

Boran et al reported that 10 of 2,200 patients (0.45%) developed exercise stress test–related ischemia and conduction abnormalities.^7^ Among these 10 patients, left anterior fascicular block (LAFB) occurred in 4 individuals, left posterior fascicular block developed in 2 patients, RBBB occurred in 2 patients, and the other 2 patients had RBBB with left axis deviation and LAFB that was progressing to LBBB.^7^ In a similar case, a patient had exercise-induced lightheadedness and syncope and was diagnosed with exercise-induced AV block during an exercise stress test.^16^ A conduction abnormality can advance to third-degree AV block, which generally warrants permanent pacemaker implantation whether the patient is symptomatic or not.^12^ Therefore, the electrophysiologic study may be reasonable to determine abnormal refractoriness of the His-Purkinje conduction system in patients with a high risk of conduction abnormalities.^17^

Based on our literature review, no clear guideline recommendation is available for exercise stress test–induced heart block management, and most of the incidences were managed differently. Bonikowske et al reviewed the Mayo Clinic Integrated Stress Center database between 2006-2010, and 40,715 tests were performed during the study period.^12^ Definite exercise-induced second-degree AV block was found in only 19 patients (0.05%; 5 females and 14 males). Of these, AV block was intermittent in 11 and persistent in 8 patients. Seven of the 8 patients required permanent pacemaker placement, and an intervention for exercise-induced AV block was not always necessary.^12^

In our case, AV dissociation with competing sinus and junctional rhythm occurred during the recovery stage and manifested with transient loss of consciousness without any sensory or motor weakness. The patient recovered with supportive measures: lying her down in the Trendelenburg position and administering IV fluids. The etiology of the patient's symptoms could be explained by a sudden drop in HR immediately after the exercise with AV asynchronization, resulting in decreased cardiac output, hypotension, and syncope. With no evidence of ischemic heart disease on the coronary angiogram and echocardiogram, the cause of the patient's chest pain was most likely uncontrolled BP. Therefore, appropriate medical optimization with losartan and nifedipine ER for better BP control was ordered. Given the presence of an isolated bradycardic event after exercise, we opted to perform additional continuous cardiac monitoring rather than placing a permanent pacemaker. We also used a loop recorder for further monitoring, and the patient was followed up in the outpatient cardiology clinic. She did not report any symptoms since her hospital discharge.

## CONCLUSION

The exercise stress test is considered a benign test and is used daily in contemporary practice. Clinicians’ awareness regarding conduction abnormalities related to the exercise stress test is pivotal to provide immediate treatment when complications occur. The evidence in the literature regarding how to manage the isolated exercise-induced AV dissociation with junctional rhythm remains unclear. Additional cardiac monitoring is a reasonable approach to observe future events to decide whether the patient would benefit from permanent pacemaker placement.

## Notes

*This article meets the Accreditation Council for Graduate Medical Education and the American Board of Medical Specialties Maintenance of Certification competencies for Patient Care and Medical Knowledge.*
